# Mean Expression of the X-Chromosome is Associated with Neuronal Density

**DOI:** 10.3389/fnins.2012.00161

**Published:** 2012-11-12

**Authors:** James T. Swingland, Pascal F. Durrenberger, Richard Reynolds, David T. Dexter, Ana Pombo, Manuel Deprez, Federico Roncaroli, Federico E. Turkheimer

**Affiliations:** ^1^Institute of Psychiatry, King’s College LondonLondon, UK; ^2^Division of Brain Sciences, Imperial College Faculty of Medicine, Hammersmith Hospital CampusLondon, UK; ^3^MRC Clinical Sciences Centre, Imperial College School of Medicine, Hammersmith Hospital CampusLondon, UK; ^4^Laboratory of Neuropathology, Department of Pathology, University Hospital of LiègeBelgium

**Keywords:** Chromowave, chromosome X, dosage compensation, wavelets, neuronal concentration

## Abstract

**Background:** Neurodegenerative diseases are characterized by key features such as loss of neurons, astrocytosis, and microglial activation/proliferation. These changes cause differences in the density of cell types between control and disease subjects, confounding results from gene expression studies. Chromosome X (ChrX) is known to be specifically important in the brain. We hypothesized the existence of a chromosomal signature of gene expression associated with the X-chromosome for neurological conditions not normally associated with that chromosome. The hypothesis was investigated using publicly available microarray datasets from studies on Parkinson’s disease, Alzheimer’s disease, and Huntington’s disease. Data were analyzed using *Chromowave*, an analytical tool for detecting spatially extended expression changes along chromosomes. To examine associations with neuronal density and astrocytosis, the expression of cell specific reporter genes was extracted. The association between these genes and the expression patterns extracted by *Chromowave* was then analyzed. Further analyses of the X:Autosome ratios for laser dissected neurons, microglia cultures and whole tissue were performed to detect cell specific differences. **Results:** We observed an extended pattern of low expression of ChrX consistent in all the neurodegenerative disease brain datasets. There was a strong correlation between mean ChrX expression and the pattern extracted from the autosomal genes representing neurons, but not with mean autosomal expression. No chromosomal patterns associated with the neuron specific genes were found on other chromosomes. The chromosomal expression pattern was not present in datasets from blood cells. The X:Autosome expression ratio was also higher in neuronal cells than in tissues with a mix of cell types. **Conclusions:** The results suggest that neurological disorders show as a reduction in mean expression of many genes along ChrX. The most likely explanation for this finding relates to the documented general up-regulation of ChrX in brain tissue which, this work suggests, occurs primarily in neurons. If validated, this cell specific ChrX expression warrants further research as understanding the biological reasons and mechanisms for this expression, may help to elucidate a connection with the development of neurodegenerative disorders.

## Introduction

In recent years several studies have used expression microarrays to investigate the molecular signature of neurodegenerative diseases and identify candidate genes relevant to their pathogenesis (Blalock et al., 2004; Hodges et al., 2006; Moran et al., 2006; Papapetropoulos et al., 2006; Scherzer et al., 2007). Irrespective of the condition, the affected brains show a progressive and irreversible nerve cell loss that increases with the severity of the disease (Hedreen and Folstein, 1995; Gómez-Isla et al., 1997; Bossers et al., 2009). Brains with neurodegenerative diseases also show reactive astrocytosis and microglial activation. Such changes result in an altered ratio of neuronal and non-neuronal mRNA and represent an important variable in gene expression studies (Oldham et al., 2008; Clarke et al., 2010).

The X-chromosome (ChrX) contains a large number of genes that are essential for brain development and function (Laumonnier et al., 2007). Several brain disorders are also associated with mutations of genes on ChrX (Oostra and Willemsen, 2002; Chadwick and Wade, 2007; Zanni and Bertini, 2011). In mammalian brains the global expression of ChrX is higher than in other tissues (Nguyen and Disteche, 2006b). Higher X-expression in brain tissue has been linked to “X dosage compensation,” a mechanism that matches the expression of X-linked genes with the expression of genes on autosomal chromosomes in organisms where sex determination depends on highly dimorphic sex chromosomes (Nguyen and Disteche, 2006a; Straub and Becker, 2007; Deng et al., 2011; Kharchenko et al., 2011). Whether all brain cells or only certain types of cells exhibit this increased expression is unknown. However it is known that varying density of different cell types in brain will impact measured expression values (Clarke et al., 2010).

Gene expression studies are an important source of data for investigating neurodegenerative disorders, however lists of differentially expressed genes often do little to improve understanding of disease mechanisms or of the underlying biology. Approaches which address microarray data analysis by looking at networks (Mitchell and Mirnics, 2012) or other system level analysis can provide a more interpretable and hence useful viewpoint (Langfelder and Horvath, 2008; Mar et al., 2011). So far, however, a systemic and important factor, spatial location, has been generally overlooked from microarray analytics (Hurst et al., 2004; Turkheimer et al., 2006). Spatial location can be important in biological systems regulated by chromatin (Anderson et al., 2008), or for variations in copy number (Turkheimer et al., 2006) and so is likely to be useful when dealing with high X-expression in the brain. *Chromowave* (Turkheimer et al., 2006; Anderson et al., 2008), is an analytical tool designed to analyze chromosomal patterns of expression variation from adjacent genes to include spatial factors into the modeling process.

In the present study, we used datasets obtained from the brains of subjects with Parkinson’s disease (PD; Moran et al., 2006; Papapetropoulos et al., 2006; Durrenberger et al., 2012), Alzheimer’s disease (AD; Blalock et al., 2004) and Huntington’s disease (HD; Hodges et al., 2006) and age matched controls to test whether similar spatial expression patterns occur in different neurological conditions. Further it was hypothesized that the patterns would mirror the changes in neuronal densities and that the patterns would arise specifically on ChrX. We used a novel approach *Chromowave* to look at the spatial expression patterns that may indicate neuronal loss. Major patterns of ChrX expression were extracted using *Chromowave*. These datasets used a range of different microarray platforms and pre-processing methods. Further expression datasets from blood cells in PD and HD were used as negative controls. The case loadings from ChrX patterns were then compared with the primary expression pattern from a series of neuronal reference genes on the autosomes as a way to infer their relationship with varying densities of neurons in the samples. Furthermore, mean ratios of X:Autosome expression were obtained from control subjects and laser dissected neurons (Dunckley et al., 2006; Zheng et al., 2010) to test whether neurons express ChrX at different levels to samples containing a mix of cell types. Primary cell culture samples of fetal microglia were also obtained from a public database to test the alternative hypothesis that imbalances in the X:Autosome ratio was due to microglial proliferation.

## Materials and Methods

### Microarray data

Data from whole tissues were obtained from online databases (mostly GEO except for the HD caudate samples from Array express) from different diseases and platforms. These datasets are summarized in Table 1, with clinical details available in the original references. Data was also obtained from blood samples for PD and HD. Blood samples were used to determine whether findings were tissue specific. These datasets were further supplemented by two publicly available datasets of laser microscopy dissected neurons. Only the control samples from these datasets were used and only those genes showing significant detection values were included. Clinical details of whole tissue samples are found in Table 2 with further details available in the original publications. Data from human fetal microglia samples from four brains were also acquired from the GEO. Microglia samples were acquired at a range of time points, and here only those from the first time point (1 h) were used. This was because the X:Autosome ratio was time dependent with these having the highest ratio.

**Table 1:** **Datasets**.

Disease	Region	Reference	Accession	Platform	Subjects
PD	Lateral nigra	Moran et al. (2006)	GSE8397	Affymetrix HG-U133A B	6 controls, 13 PD*
PD	Medial nigra	Moran et al. (2006)	GSE8397	Affymetrix HG-U133A B	5 controls, 13 PD^#^
PD	Substantia nigra	Papapetropoulos et al. (2006)	GSE7621	Affymetrix HG-U133 plus 2	9 controls, 16 PD
PD	Substantia nigra	Durrenberger et al. (2012)	GSE26927	Illumina humanRef8 v2.0	7 controls, 12 PD
AD	Hippocampus	Blalock et al. (2004)	GSE1297	Affymetrix HGU133A	8 controls, 18 AD
HD	Caudate	Hodges et al. (2006)	E-AFMX-6	Affymetrix HG-U133A B	11 controls, 17 HD
HD	blood	Hodges et al. (2006)	GSE1751	Affymetrix HGU133A	12 controls, 14 HD
PD	blood	Scherzer et al. (2007)	GSE6613	Affymetrix HGU133A	21 controls, 50 PD
controls	Substantia nigra neurons	Zheng et al. (2010)	GSE20141	Affymetrix HG-U133 plus 2	8 controls
controls	Entorhinal cortex neurons	Dunckley et al. (2006)	GSE4757	Affymetrix HG-U133 plus 2	10 controls
controls	Human fetal microglia	Rock et al. (2005)	GSE1432	Affymetrix HGU133A	4 controls

*One control and one patient were removed due to multiple sclerosis and AD respectively.

*^#^One control was removed due to multiple sclerosis. Two patients were removed one due to AD the other was a juvenile genetic case*.

**Table 2:** **Clinical details for each dataset obtained from original publications**.

Dataset	Age (yrs) controls	Age (yrs) patients	Duration of illness	PMI controls	PMI patients	Tissue pH (controls)	Tissue pH (patients)
PD medial nigra	69.3 ± 4.9	81.4 ± 1.6	13.2 ± 2.5	15.6 ± 5	12.9 ± 1.9	6.5 ± 0.13	6.3 ± 0.23
PD lateral	65.6 ± 5.2	82.3 ± 1.7	13.3 ± 1.4	12 ± 4.6	14.6 ± 3.1	6.4 ± 0.4	6.45 ± 0.08
Whole Nigra	78.26 ± 11.4	75 ± 7.4	12.9 ± 6.9	7.8 ± 4.1	6.8 ± 4.8	6.3 ± 0.3	6.2 ± 0.2
Illumina	64.5 ± 5.8	81.5 ± 1.3	13 ± 1.9	27.94 ± 4.84	12.2 ± 1.91	NA	6.82 ± 0.2
AD	85.3 ± 2.7	85.7 ± 2.6	NA	2.6 ± 0.2	3.2 ± 0.4	NA	NA
HD	53 ± 20	53 ± 16	NA	NA	NA	NA	NA

Neuronal and astrocytic reporter genes were selected from a transcriptome database (Cahoy et al., 2008). Only genes with fold change >20 in the database were included, with those on ChrX excluded *a priori* to avoid biasing for that chromosome. All probes representing any of these genes in the dataset were included.

### Microarray data pre-processing

The Data from the lateral and medial substantia nigra were already normalized and log2 transformed, as described in the original study (Moran et al., 2006). For the Illumina dataset, normalization was conducted using the Rosetta error models available in the Rosetta Resolver^®^ system (Rosetta Biosoftware, Seattle, WA, USA; Weng et al., 2006). Values were then log2 transformed. Data from other datasets were normalized by dividing intensities by the median value of those genes presenting with positive detection (present call *P* in Affymetrix notation). Values were then log2 transformed.

### Chromowave analysis of gene expression

Data were analyzed with *Chromowave* (Turkheimer et al., 2006; Anderson et al., 2008), written in MATLAB (The Mathworks, Inc., Natick, MA, USA). *Chromowave* first mapped the probes from the datasets to their corresponding chromosomal location and then applied the wavelet transform to their spatial distribution, converting the expression values into wavelet coefficients. Each wavelet coefficient represents the average value of adjacent probes at different length scales (2-4-8-16… adjacent probes depending on the scale) in each specific location on a chromosome and can be manipulated using standard statistical methodologies to extract coherent spatial patterns in the data (Turkheimer et al., 2006).

The wavelet transform represents the data in a different form which emphasizes the similarity in expression between groups of adjacent probes. Transforming the data into wavelet space amplifies groups of adjacent probes with similar expression behavior so that they have a larger effect on the analysis than individual probes when analyzed independently. For example if a large group of adjacent probes were more highly expressed in patients than in controls this may result in a significant difference in the wavelet representation, even though none of the probes would individually demonstrate a significant signal. The wavelet methodology analyzes the data through a range of different scales (from adjacent pairs of probes to whole chromosomes) and is therefore suitable for detecting relatively small events incorporating a handful of probes to larger events up to whole chromosomes or genomes. The wavelet transform is widely used in many fields, including gene analysis (Lio, 2003). *Chromowave* has been validated in previous publications for the detection of expression differences caused by copy number variation (Turkheimer et al., 2006) and disruption caused by Huntington’s disease (Anderson et al., 2008).

In *Chromowave*, statistical analysis is performed on the wavelet coefficients by application of the singular value decomposition (SVD), a proven technique for the reduction of data dimensionality also used in microarray data analysis (Alter et al., 2000). SVD factorizes the data into a set of expression patterns (equivalent to principle components) and case loadings. Case loadings are a series of values each representing the strength of the detected spatial pattern for each individual subject. As each subject is represented by one case loading, these values can be analyzed using standard statistical techniques, such as regression models and *t*-tests. Performing the analysis in wavelet space instead of on the probe sets, identifies the major spatial patterns of gene expression (Turkheimer et al., 2006), rather than the functional expression patterns of individual probes.

In this work, *Chromowave* was applied to X-linked probes for each dataset and the largest component from chromosome X was extracted. Values which were inferred to represent noise (Turkheimer et al., 2006) were removed and the remaining coefficients were transformed back into differential expression values and plotted against their genomic location. The resulting profiles are therefore smoothed representations of the spatial variation in expression plotted against their chromosomal position. The case loadings for each subject associated with the X related patterns were correlated with the expression of a series of “reporter genes” indicative of neuronal, astrocytic and microglial mRNA expression. Other disease related variables were also investigated.

### Extraction of patterns from reporter genes

Genes representative of neurons and astrocytes were selected from a transcriptome database (Cahoy et al., 2008). Only genes with fold change >20 in the database publication were chosen, except those on ChrX which were excluded *a priori* to avoid spurious association. The expression of all the probes representing these genes in each dataset were extracted, logged, and analyzed using SVD. This allowed for the extraction of the primary expression pattern from these genes. This is a more robust method to summarize the data than a simple average, though averages were subsequently used to verify the direction of association. The correlation coefficients between the primary patterns extracted here and the case loadings obtained from chromosomal expression were then calculated.

### Statistical analysis

Statistical analysis was performed in MATLAB R2011a (The Mathworks, Inc., Natick, MA, USA), using parametric methods (Pearson correlation coefficients, Student *t*-tests, both two-tailed).

## Results

### X-expression neurodegeneration and aging

**Figure 1:**
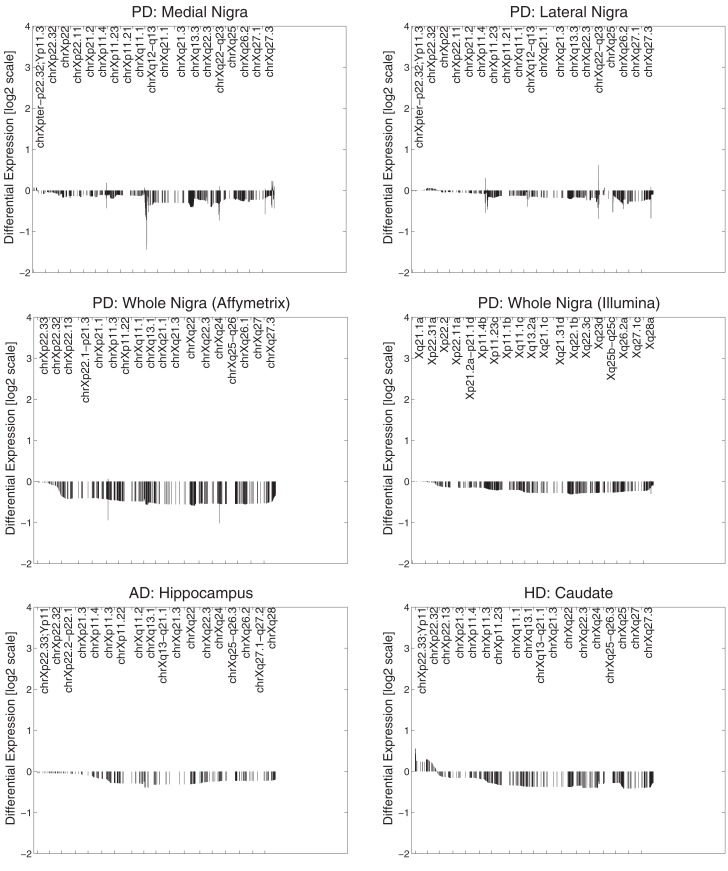
**Primary expression pattern from ChrX extracted via *Chromowave* from all brain samples**. The case loadings for all patterns correlated significantly with the activity of the “neuronal reporter genes” used. Negative differential expression values indicate low Chromosome X-expression associated with reduced neuronal reporter gene expression, positive values indicated increased expression. Reduced expression is also associated with neurological disease. The patterns demonstrate reduced gene expression over the majority of ChrX with the exception of the *p*-end.

All datasets from whole tissue brain samples demonstrated similar, patterns of ChrX expression that extended through the whole chromosome with the exception of the telomeric *p*-region (see Figure 1). Patterns were reproducible despite the variety of platforms, protocols, and normalization methods used. All case loadings from ChrX correlated with the patterns of expression extracted from the autosomal genes representing neuronal density (Table 3) with lower expression of those X-genes in the profile associated with lower expression of the neuronal reporter genes. In a few datasets significant correlations were observed with the astrocytic reporter gene-set (Table 4) but in these instances the variance explained was lower than the one explained by the neuronal gene-set. Stepwise multiple regression of these datasets indicated that astrocytic reporter genes did not add significant extra information to that provided by the neuronal reporter genes.

**Table 3:** **Correlation of X-pattern from whole tissue with neuronal reporter genes**.

Disease	Tissue region	Platform	Correlation
PD	Lateral nigra	Affymetrix HGU133A and B	*r* = 0.82, *p* = 0.0006
PD	Medial nigra	Affymetrix HGU133A and B	*r* = 0.75, *p* = 0.0002
PD	Substantia nigra	Affymetrix HGU133 plus 2	*r* = 0.89, *p* = 3 × 10^−9^
PD	Substantia nigra	Illumina HumanRef8 v2	*r* = 0.86, *p* = 1 × 10^−6^
AD	Hippocampus	Affymetrix HGU133A	*r* = 0.8, *p* = 1 × 10^−6^
HD	Caudate	Affymetrix HGU133A and B	*r* = 0.92, *p* = 2 × 10^−12^

**Table 4:** **Correlation of X-pattern from whole tissue with astrocytic reporter genes**.

Disease	Tissue region	Platform	Correlation
PD	Lateral nigra	Affymetrix HGU133A and B	*r* = 0.04, *p* = 0.9
PD	Medial nigra	Affymetrix HGU133A and B	*r *= 0.3, *p* = 0.3
PD	Substantia nigra	Affymetrix HGU133 plus 2	*r *= 0.3, *p* = 0.1
PD	Substantia nigra	Illumina HumanRef8 v2	*r* = 0.0004, *p* > 0.99
AD	Hippocampus	Affymetrix HGU133A	*r* = 0.48, *p* = 0.01
HD	Caudate	Affymetrix HGU133A and B	*r *= 0.67, *p* = 0.0001

**Figure 2:**
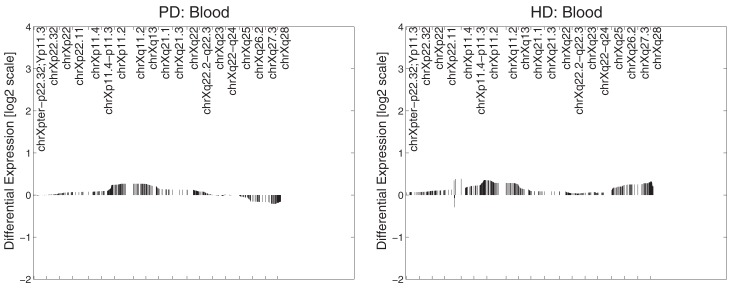
**Primary expression pattern from PD blood (left) and HD blood (right) datasets**. Profiles were not associated with neuronal reporter genes and are visually different from each other and those obtained from brain tissue.

In PD brain tissue, ChrX case loadings differed between patients and controls if female subjects were removed (*p* = 0.047, 0.00004, 0.007, and 0.002 respectively). In the HD caudate the case loadings differed significantly between patients and controls (*p* = 6 × 10^−5^). Removing gender did not produce significance in other datasets and no comparisons of genders were made in any dataset (due to small numbers of subjects). Patterns derived from brain tissues were not reproduced in datasets derived from blood tissues (Figure 2).

Loss of neurons is also generally associated with natural aging and hence the case loadings from the largest control group (caudate samples) were correlated against age. The results showed a trend correlation (*r *= 0.79, *p* = 0.08) of decreasing X-expression (of the genes in the pattern) with age. In the patient groups there was significant association of case loadings with duration of illness in the Illumina dataset (*r* = 0.6, *p* = 0.04) but not in the medial nigra (*r* = 0.34 *p* = 0.3) or the lateral nigra (*r* = 0.22 *p* = 0.6) datasets. No significant associations were found in these datasets between the case loadings and post-mortem interval, pH or brain weight.

### Autosomes and neurodegeneration

Application of *Chromowave* to the autosomal probes in the datasets above retrieved patterns that were associated with neurodegeneration in AD (*r* = 0.7, *p* = 0.00001) and HD (*r* = 0.6, *p* = 0.0006), but not in the PD datasets, however these patterns varied widely across datasets. Results such as these are likely to represent disease specific findings or false positives, and as such are outside the scope of this paper.

### ChrX:Autosome ratio for laser micro-dissected neurons and whole tissue

To investigate whether neurons have higher mean expression of ChrX than other brain cell types, publicly available neuronal data acquired through laser dissection microscopy was used. The X:Autosome ratio for each laser dissected sample was compared with the ratio for control subjects for each whole tissue dataset. The two sets of dissected neurons had higher mean X:Autosome ratios than the control cases from the whole tissue samples. The *p*-values for each comparison can be found in Table 5.

**Table 5:** **Higher X:Autosome ratio for laser dissected neurons than whole tissues**.

Neuron sample location	Array type	Whole tissue location	Array type	*p*-value
Substantia nigra	Affymetrix HGU133 plus 2	Substantia nigra	Affymetrix HGU133 plus 2	*p* = 5 × 10^−5^
Substantia nigra	Affymetrix HGU133 plus 2	Medial substantia nigra	Affymetrix HGU133A and B	*p* = 7 × 10^−5^
Substantia nigra	Affymetrix HGU133 plus 2	Lateral substantia nigra	Affymetrix HGU133A and B	*p* = 7 × 10^−5^
Substantia nigra	Affymetrix HGU133 plus 2	Substantia nigra	Illumina HumanRef8 v2	*p* = 4 × 10^−5^
Substantia nigra	Affymetrix HGU133 plus 2	Hippocampus	Affymetrix HGU133A	*p* = 1 × 10^−4^
Substantia nigra	Affymetrix HGU133 plus 2	Caudate	Affymetrix HGU133A and B	*p* = 6 × 10^−5^
Entorhinal cortex	Affymetrix HGU133 plus 2	Substantia nigra	Affymetrix HGU133 plus 2	*p* = 5 × 10^−3^
Entorhinal cortex	Affymetrix HGU133 plus 2	Medial substantia nigra	Affymetrix HGU133A and B	*p* = 4 × 10^−3^
Entorhinal cortex	Affymetrix HGU133 plus 2	Lateral substantia nigra	Affymetrix HGU133A and B	*p* = 7 × 10^−3^
Entorhinal cortex	Affymetrix HGU133 plus 2	Substantia nigra	Illumina HumanRef8 v2	*p* = 3 × 10^−5^
Entorhinal cortex	Affymetrix HGU133 plus 2	Hippocampus	Affymetrix HGU133A	*p* = 6 × 10^−2^
Entorhinal cortex	Affymetrix HGU133 plus 2	Caudate	Affymetrix HGU133A and B	*p* = 5 × 10^−5^

### ChrX:Autosome ratio for microglia and whole tissue

To test the alternative hypothesis that it is the X:Autosome ratio in microglia driving the chromosome X results, four publicly available microglial control subjects were used. The X:Autosome ratio for these four samples was 1 ± 0.01, the expected ratio for non-brain tissue. The X:Autosome ratio for these four samples was significantly lower than controls from all datasets of laser dissected neurons and whole tissue samples (except the substantia nigra samples measured using the Illumina array). The *p*-values for each comparison are included in Table 6.

**Table 6:** **Lower X:Autosome ratio for microglia than neurons and whole tissues**.

Sample location	Array type	*p*-value
Laser dissected neurons (substantia nigra)	Affymetrix HGU133 plus 2	4 × 10^−5^
Laser dissected neurons (Entorhinal cortex)	Affymetrix HGU133 plus 2	2 × 10^−5^
Lateral substantia nigra	Affymetrix HGU133A and B	6 × 10^−3^
Medial substantia nigra	Affymetrix HGU133A and B	2 × 10^−3^
Substantia nigra	Affymetrix HGU133 plus 2	5 × 10^−3^
Substantia nigra	Illumina HumanRef8 v2	0.2
Hippocampus	Affymetrix HGU133A	6 × 10^−7^
Caudate	Affymetrix HGU133A and B	4 × 10^−7^

## Discussion

The first set of our results demonstrated a reduced chromosome X-expression in brain (with the exclusion of the far telomeric Xp region) associated with neurodegenerative disorders. The pattern of low expression was strongly associated with lower expression of the neuronal reporter genes in all brain datasets. There was a significant association with the astrocytic reporter genes in two datasets (AD caudate and HD hippocampus) which is likely due to the correlation between astrocytosis and loss of neurons. The association was less than that with the neuronal reporter genes and application of stepwise regression indicated that the association did not explain significant extra variance. Neuronal reporter genes were not associated with autosomal patterns or mean autosomal expression. To test further whether reduction in expression in neurodegeneration was due to neuronal loss in the samples, the available mRNA data on laser dissected neurons were analyzed and we showed that laser dissected neurons had higher X:Autosome expression ratios than whole brain. Analysis of microglia samples suggested that microglia have lower ratios than whole brain tissues (~1) and therefore do not contribute to the high X-expression in brain. In short, the strong association of ChrX reduction with the neuronal reference genes and the ancillary results on laser extracted neurons and microglia cells suggest that this pattern of reduced expression reflects the changes in cell populations that occur in neurodegenerative disorders. These results have been reproduced consistently in multiple datasets, diseases and across different array platforms, sampling protocols, and normalization methods. Chromosome specificity was striking and the pattern of expression extended through the whole of ChrX with the exception of the far *p*-end. The simplest explanation for these results is that the high X:autosome ratio in brain (Nguyen and Disteche, 2006b) is caused by high X-expression in neurons. These presumably show higher expression of the genes located in the *Chromowave* expression pattern than other cell types or tissues. As neurons die off this causes the average expression of these genes to reduce, creating the observed pattern and implying a shift in the X:autosome ratio. Alternatively it could be that microglia show abnormally low X:autosome ratios, however this is less likely as the X:Autosome ratio for these cells is ∼1, which is the expected ratio for non-brain tissue. That microglia show lower ratios than whole tissue is explained by noting that whole tissue contains neurons and so show higher ratios than tissues which do not contain neurons.

Interestingly, the pattern obtained by *Chromowave* from ChrX resembles the pattern reported in ChrX-inactivation. X-inactivation is an epigenetic event in mammalian females that results in the transcriptional silencing of one X-chromosome. In X-inactivation not all X-linked genes are repressed and, in humans, the proportion of genes on the X-chromosome that escapes inactivation is more than 15%, mostly located on the *p*-end (Brown and Greally, 2003). One can therefore speculate on a link between epigenetic mechanisms of X-inactivation and the increased activation of X-linked genes in neurons and also on the reasons for this higher activity. As well as brain genes, the X-chromosome also has a large number of genes related to the immune system. X-chromosome dosage has been linked to autoimmune disorders (Libert et al., 2010; Svyryd et al., 2012) while immune reactivity and neuroinflammation have long been associated with neurological conditions (Fang et al., 2012; Medeiros et al., 2012; Saing et al., 2012). This makes it tempting to speculate on possible reasons for the high X-expression in neurons and possible links with disease progression, however much further work would be needed to investigate such possibilities. In any case however, the results contained in this manuscript suggest that, studies on the role of X-linked probes in neurodegenerative disorders must be tightly controlled for cell numbers.

One further point of note in this report is the consistent observation that when female subjects were removed the X-pattern was significantly different between controls and PD subjects. In our view, this does not mean that females show significantly different X-loadings to males, or differ in their response to neuronal loss. Possibly males show a larger distinction between controls and PD patients due to female subjects having a more benign phenotype with milder degeneration (Haaxma et al., 2007). In this case, further studies with greater sample sizes are warranted.

Samples used in this investigation came from a range of diseases, though more were taken from PD than other neurological conditions. Since results were similar in all datasets, PD and non-PD, it is unlikely that this has added any meaningful bias to the analysis. Additionally although there are clear similarities between the profiles obtained from these different conditions the analysis does not rule out disease specific changes occurring on ChrX and in the autosomes. However any disease specific results are outside the scope of this paper and no autosomal pattern was replicated in the datasets.

It is worth noting that *Chromowave* results are made up of many hundreds of probes. Individually these probes have only weak (mostly non-significant) associations with the neuronal density and so standard single genes approaches are unlikely to replicate these findings. Taking the average of many probes does reproduce the association, however it does not provide the pattern *Chromowave* provided, and is not data driven, requiring a hypothesis about chromosome wide expression changes. This helps to demonstrate the usefulness of the *Chromowave* approach when applied to situations involving spatially adjacent genes.

In conclusion, we observed a spatial pattern of low ChrX expression associated with a range of neurological diseases. This pattern was strongly associated with the activity of a set of autosomal “neuronal reporter” genes. This result most likely reflects high X-expression in neurons, a view supported by the higher X:autosome ratio found in micro-dissected tissue. This likely relates to the previously known high X-expression in brain tissues which this suggests it occurs largely in neurons. The spatial pattern of the X-expression more interestingly suggests a link between X dosage compensation, ChrX up-regulation in brain and the development and function of neuronal cells. Assuming the findings and interpretations here can be confirmed, it is also to our knowledge the first time that a specific cell type has been linked to spatially coherent transcription.

## Conflict of Interest Statement

The authors declare that the research was conducted in the absence of any commercial or financial relationships that could be construed as a potential conflict of interest.

## Abbreviations

AD, Alzheimer’s disease; ChrX, chromosome X; GEO, gene expression omnibus; HD, Huntington’s disease; PD, Parkinson’s disease; SVD, singular value decomposition.
